# MMR Vaccine and COVID-19: Measles Protein Homology May Contribute to Cross-Reactivity or to Complement Activation Protection

**DOI:** 10.1128/mBio.03447-20

**Published:** 2021-02-02

**Authors:** Ekaterina Marakasova, Ancha Baranova

**Affiliations:** a School of Systems Biology, George Mason University (GMU), Fairfax, Virginia, USA; Albert Einstein College of Medicine

## LETTER

**FIG 1:**
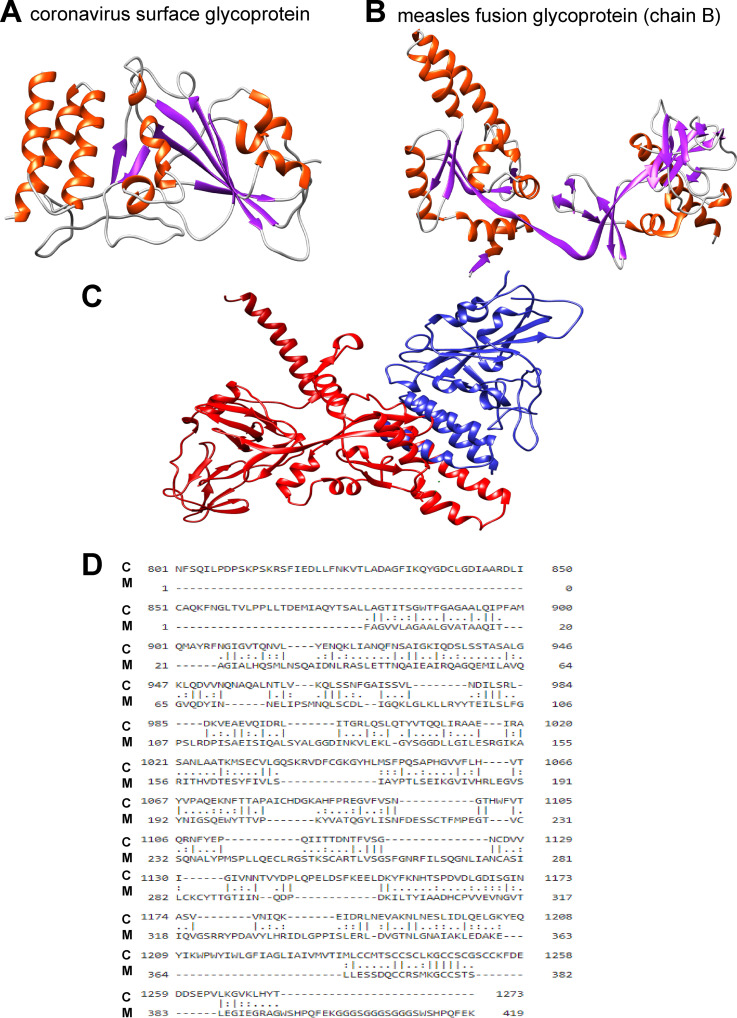
Structural characteristics of coronavirus surface glycoprotein and measles fusion glycoprotein. (A) Protein structure of coronavirus surface glycoprotein in complex with LCB1. The PDB accession no. 7JZU (12) was visualized by Chimera version 1.13.1 (13) and colored by secondary structure as follows: red, helix; purple, strand; gray, coil. (B) Protein structure of measles fusion glycoprotein (chain B). The PDB (5YXW [14]) for measles fusion glycoprotein was visualized by Chimera version 1.13.1 (13) and colored by secondary structure as follows: red, helix; purple, strand; gray, coil. (C) Aligned protein structures by Chimera version 1.13.1 (13). The coronavirus surface glycoprotein (7JZU [12]) (blue) and measles virus fusion glycoprotein chain B (5YXW [14]) (red) are shown. (D) Sequence comparison between coronavirus surface glycoprotein and measles virus fusion glycoprotein (chain B). Pairwise sequence analysis of coronavirus surface glycoprotein (NCBI accession no. YP_009724390.1) and measles fusion glycoprotein (5YXW_B) was performed by Emboss Needle version 6.6.0 (15). Abbreviations: C, coronavirus; M, measles. Full sequence identity: 93/1,393 (6.7%); # similarity: 152/1,393 (10.9%). Solvent accessibility for aligned sequences was calculated by RaptorX-Property (16) as follows: 45% exposed, 23% medium, and 30% buried for coronavirus surface glycoprotein and 41% exposed, 28% medium, and 30% buried for measles fusion glycoprotein (chain B).

A recently published study by Jeffrey E. Gold et al. (1) presents data that strongly suggest that measles-mumps-rubella (MMR) vaccination negatively correlates with the severity of coronavirus disease 2019 (COVID-19)-related symptoms. Another study by Alba Grifoni et al. (2) demonstrated that antibody titers to spike protein in some unexposed to severe acute respiratory syndrome coronavirus 2 (SARS-CoV-2) subjects may reach substantial levels, thus suggesting preexisting immunity to the coronavirus. This preexisting immunity may be due to the cross-reactivity with other antigens, for example the ones resulting from previous immunizations. Other studies reported that COVID-19 mortality is higher in countries where Mycobacterium bovis BCG vaccination is not routinely administered (3). Why previously received MMR vaccine may aid in reducing the severity of coronavirus infection symptoms is not clear, but it is tempting to speculate that one or more of the MMR components may be structurally similar to SARS-CoV epitopes recognized by the immune system and may contribute to cross-immunity. Hence, we performed homology analysis between the receptor binding domain (RBD) of the spike protein and the nucleocapsid protein of SARS-CoV-2 to measles, mumps, and rubella proteomes using BLAST (4). A similarity between the RBD of the surface glycoprotein of COVID-causing coronavirus and the measles fusion glycoprotein (chain B) was evident (Fig. 1).

The fusion protein of the measles virus is necessary for virus-cell membrane merging and subsequent injection of its ribonucleocapsid complex into the host cell cytosol. More specifically, in fusion glycoprotein (chain B) of attenuated strains of the measles virus of MMR vaccine interacts with the host cell surface receptors, including CD46 (5). Immunogenicity of the fusion glycoprotein is well-known, as it is reported as an effective target for serological responses (6). Similarly, coronavirus RBD is also recognized as an epitope for immune response (7). Our findings support the hypothesis that the chain B of the fusion protein of the measles virus may play a role in anti-SARS-CoV-2 immunological responses by its cross-reaction with RBD protein of the COVID-19-causing virus. An alternative explanation to the MMR-induced alleviation of coronavirus infection is that the RBD of coronaviral S protein may weakly engage the receptor for measles virus CD46, a complement regulatory molecule, also known as membrane cofactor protein MCP (8). It is tempting to speculate that anti-measles virus antibodies may bind SARS-CoV-2 in a way that prevents it from interacting with CD46 receptor, which normally protects the cells against complement-mediated cell lysis and, therefore, alleviates COVID-related abnormal activation of the complement posed in some recent studies (9–11). Notably, only attenuated measles vaccine strains recognize CD46, while wild-type disease-causing virus employs other entry (8), possibly explaining why the anti-COVID protective effects are detected after MMR vaccination but not as an aftermath of the natural measles disease (8). In our opinion, the findings described above warrant investigation.
